# Peritoneal Dialysis Care in Mainland China: Nationwide Survey

**DOI:** 10.2196/39568

**Published:** 2023-03-14

**Authors:** Ping Li, Xueying Cao, Weicen Liu, Delong Zhao, Sai Pan, Xuefeng Sun, Guangyan Cai, Jianhui Zhou, Xiangmei Chen

**Affiliations:** 1 Department of Nephrology First Medical Center of Chinese People's Liberation Army General Hospital Beijing China; 2 Nephrology Institute of the Chinese People's Liberation Army First Medical Center of Chinese People's Liberation Army General Hospital Beijing China; 3 State Key Laboratory of Kidney Diseases First Medical Center of Chinese People's Liberation Army General Hospital Beijing China; 4 National Clinical Research Center for Kidney Diseases First Medical Center of Chinese People's Liberation Army General Hospital Beijing China; 5 Beijing Key Laboratory of Kidney Disease Research First Medical Center of Chinese People's Liberation Army General Hospital Beijing China

**Keywords:** renal replacement therapy, peritoneal dialysis, medical quality, tertiary hospital, secondary hospital, China

## Abstract

**Background:**

Peritoneal dialysis (PD) care in mainland China has been progressing in the past 10 years.

**Objective:**

To complement information from the dialysis registry, a large-scale nationwide survey was conducted to investigate the current infrastructure and management of PD care at hospitals of different tiers.

**Methods:**

A web-based multiple-choice questionnaire was distributed through the National Center for Nephrology Medical Quality Management and Control to PD centers of secondary and tertiary hospitals in October 2020. The 2-part survey collected the information of PD centers and the clinical management of patients on PD. A total of 788 effective surveys from 746 hospitals were voluntarily returned, and data were extracted and analyzed.

**Results:**

The effective survey data covered 101,537 patients on PD, with 95% (96,460/101,537) in the tertiary hospitals. The median number of patients per PD center was 60 (IQR 21-152); this number was 32 (IQR 8-65) and 70 (IQR 27-192) for secondary and tertiary hospitals, respectively. There was a discrepancy in the availability of designated physical areas for different functions of PD care between the secondary and tertiary hospitals. The proportion of tertiary hospitals with PD training (*P*=.01), storage (*P*=.09), and procedure area (*P*<.001) was higher compared to secondary hospitals. PD catheter placement was performed in 96% (608/631) of the PD centers in tertiary hospitals, which was significantly higher compared to 86% (99/115) in secondary hospitals (*P*<.001). Automated PD was available in 55% (347/631) of the tertiary hospitals, which was significantly higher than that in secondary hospitals (37/115, 32%) according to the survey (*P*<.001). The most commonly performed PD module was continuous ambulatory peritoneal dialysis (772/788, 98%), followed by intermittent peritoneal dialysis (543/788, 69%). The overall reported nocturnal intermittent peritoneal dialysis was 31% (244/788); it was 28% (220/788) for continuous cycling peritoneal dialysis and 15% (118/788) for tidal peritoneal dialysis. Comparisons between the secondary and tertiary hospitals revealed no significant differences in prophylactic antibiotic use for PD catheter placement and therapeutic use for peritonitis. The first peritoneal equilibrium test was conducted in 58% (454/788) of patients at 4-6 weeks after initiation of PD, and 91% (718/788) reported at least one peritoneal equilibrium test per year. Overall, 79% (570/722) and 65% (469/722) of PD centers performed assessment for dialysis adequacy and residual kidney function, respectively; and 87% (685/788) of patients on PD were followed every 1 to 3 months for laboratory and auxiliary examinations.

**Conclusions:**

This national survey reflects the current status and disparities of PD center management in mainland China. The study results suggest that the PD care needs to be more conveniently accessible in secondary hospitals, and quality management and staff training in secondary hospitals are still in high demand.

## Introduction

It was not until the late 1990s that peritoneal dialysis (PD) started to grow rapidly in academic hospitals in large cities of mainland China [1]. Although those top PD centers gained significantly improved clinical outcomes in the late 2000s, the accumulated experiences were shared through published materials of standard operating procedure (SOP) for PD management [2] and through organized training materials [3] and courses from 2011 to 2012.

In China, the pyramid of the health care system is constructed by 3 tiers of hospitals and community convenient clinics. There were about 3000 tertiary hospitals and more than 10,000 secondary hospitals in China according to the 2020 China Health Care Development Statistics [4]. The tertiary hospitals are the top-level hospitals usually located in regional capitals, serving as teaching hospitals as well as medical care providers to manage complex and incurable diseases. The primary hospitals and clinics are often the service providers for long-term disease follow-up, general prescription renewal, and preventive care. Health care workers at tertiary hospitals and in large cities tended to have better exposure to up-to-date medical knowledge, clinical management concept, and more training opportunities for advanced procedures. More patients in end-stage kidney disease (ESKD) choose PD as their renal replacement therapy due to continuing increase in the prevalence and incidence of ESKD [5,6], average educational level of the patients, availability and accessibility of PD resources, and better policy support. In the recent 10 years, PD service is available not only in leading tertiary hospitals but also in secondary hospitals, which has raised a concern about the sufficient governance of PD care quality.

In mainland China, there has been a nationwide registry for dialysis—Chinese National Renal Data System (CNRDS) [7-9]—along with some independent provincial-level databases [10-12] that mostly record clinical and socioeconomic outcomes. Detailed facility infrastructure data, especially for PD centers, are not available in these databases. Although some data related to the concept and behavior of PD care providers (such as the common follow-up parameters, check-up schedule, and the use of medicine for comorbidities and peritonitis) are included in the dialysis registries, these data are largely not spontaneously reported. In addition, some of the information about the reasons that hindered PD as the choice for renal replacement therapy are not collected by the registries. Moreover, previously reported results from large-scale, real-world studies or analysis of registry data did not include information from the recent 2 years [12-14]. Thus, there is a strong need to have another way to collect real-world PD care data and elicit relevant information for better PD care in the future.

To complement a comprehensive picture of current PD practice in mainland China, a national survey was designed focusing on hospital infrastructure for PD as well as conceptual modalities and goals for management of patients on PD. By October 2020, the time when the questionnaires were distributed, data from CNRDS showed that there were about 1000 hospitals offering PD service, with only few in primary hospitals.

## Methods

### Participants

From September to October 2020, according to the information in the CNRDS, renal divisions from tertiary and secondary hospitals known to have or likely to have PD programs were invited to the web-based survey. Through the National Center for Health Care Quality Management in Nephrology Diseases, the invitation QR code and questionnaire instruction pack was first sent to each provincial Center for Health Care Quality Management in Nephrology Diseases and then distributed to physicians whose practice field was PD. Survey results were voluntarily submitted by participating centers and practitioners.

### Survey Design

A multiple-choice type of questionnaire was designed and tested in a pilot group before being distributed to the target respondents. Detailed questions included in the survey are listed in Table 1. The questionnaire consisted of 2 parts. The first part collected the information of the hospital or PD center, including the location and health care level of the facility (secondary or tertiary), availability of nephrology and dialysis care resources, regulatory rules on PD, number of current patients on PD, and the types of PD performed.

The second part of the survey addressed practice concepts or the current situation of clinical management for patients on PD. The questions covered PD catheter placement and care; use of peritoneal equilibrium test (PET); regular follow-up schedule and items; as well as management of common complications, including peritonitis, anemia, and hypertension.

**Table 1:** Survey items listed in the questionnaire for peritoneal dialysis (PD).

Category	Item
Facility-related survey items	Name and location of the hospital Administrative ranking of the hospital (secondary hospital or tertiary hospital) Number of current patients on PD Types of PD performed in your center Diseases treated with PD in your center Common situations hindering you from choosing PD Functional areas designated for PD Does your center register patients on PD in the CNRDS^a^ [15]? Is PD catheter placement performed in your center? Do you perform PD catheter implantation surgery?
Concept-related survey items	Antibiotic medicine routinely given right after PD catheter placement Maximal interval for change of external connecting tube Do you prescribe or perform peritoneal equilibrium test (PET)? Time to administer the first PET Frequency of repeat PETs Common follow-up interval for patients on PD Laboratory tests and examinations administered for PD follow-up Antibiotics for treatment of peritonitis Common hemoglobin level of patients on PD in your center Use of antihypertensive drugs, number of drugs, and estimated percentage of patients

^a^CNRD: Chinese National Renal Data System.

### Statistical Analysis

Descriptive statistical analysis was performed to evaluate PD items in the survey, and median (IQR) or proportion (frequency) were reported for continuous or categorical variables, as appropriate. Comparisons by health care level of the facility were conducted using Pearson chi-square and Wilcoxon tests for categorical and continuous variables, respectively, along with figures. R (version 4.03; R Core Team) and GraphPad Prism (version 8.0; GraphPad Software Inc) were used for data analysis and figures.

### Ethical Considerations

This work was carried out under the research program of National Health Care Quality Management in Nephrology Diseases of Chinese People’s Liberation Army General Hospital. The research protocol was reviewed and exempted by the institutional review board of Chinese People’s Liberation Army General Hospital. There was no collection of individual and identifiable patient data for the survey. The original informed consent from each participating hospital allows for secondary data analysis without additional consent. All questionnaires were voluntarily responded without compensation.

## Results

### Participating PD Centers and Patients

**Figure 1:**
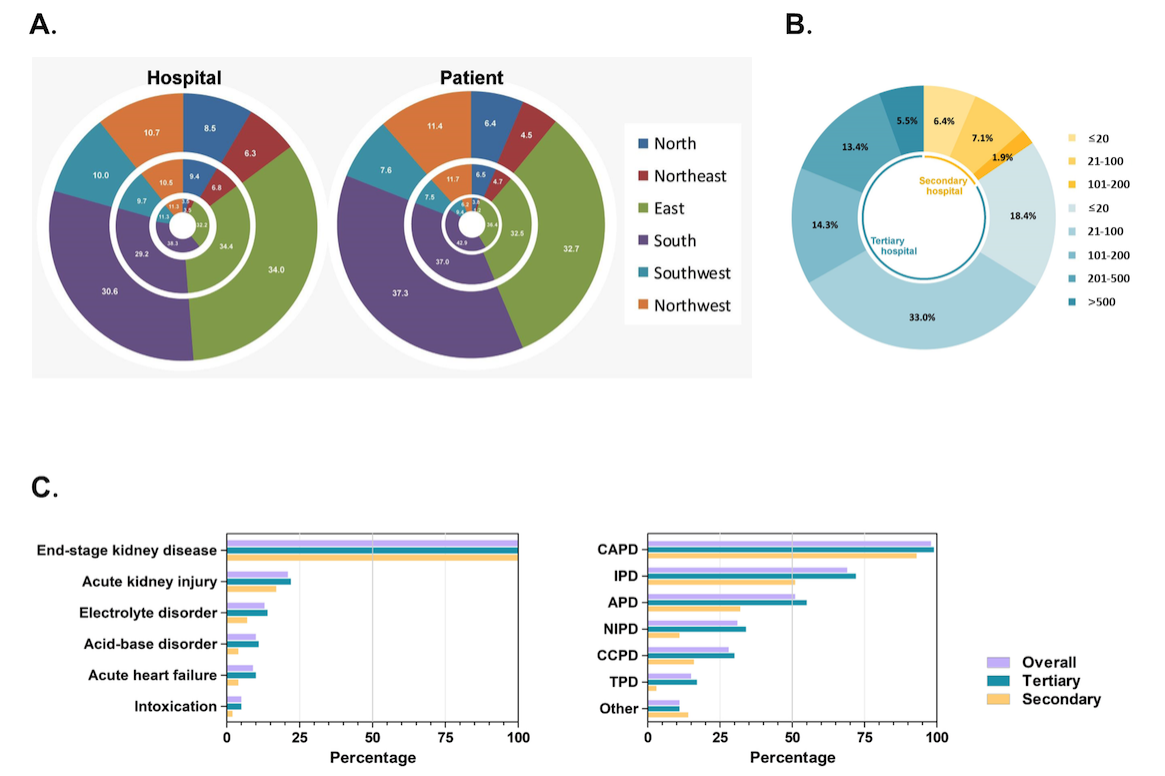
Characteristics of hospitals and patients on peritoneal dialysis (PD) covered by the survey. (A) Proportions (%) of participated hospitals and patients in different regions of mainland China; inner ring: secondary hospitals; middle ring: tertiary hospitals; and outer ring: all hospitals. (B) Proportion of participated hospitals with different number of patients on peritoneal dialysis (PD). (C) Proportions of diseases treated with PD and the types of PD performed. APD: automated PD; CAPD: continuous ambulatory PD; CCPD: continuous cycling PD; IPD: intermittent PD; NIPD: nocturnal IPD; TPD: tidal PD.

A total of 788 surveys from 746 hospitals located in all 31 provincial-level areas (ie, province, autonomous prefecture, or municipality directly under the central government) in mainland China were returned. These 746 centers covered 101,537 patients on PD, with 95% (96460/101,537) of them in the tertiary hospitals. Collected data were further grouped into 6 socioeconomic administrative areas (ie, North, Northeast, East, South, Southwest, and Northwest China); proportions of the hospitals and patients from each area are shown in Figure 1A. Overall, the median patient number per PD center was 60 (IQR 21-152), that is, 70 (IQR 26-192) for tertiary hospitals and 32 (IQR 8-65) for secondary hospitals (Figure 1B). There was no significant regional difference in the distribution of PD centers in terms of patient size. Among the 746 centers, 10 centers did not treat patients on PD, 6 (60%) of which were tertiary hospitals; however, among the 722 centers providing PD care, 611 (85%) were tertiary hospitals. All these indicate that tertiary hospitals are the primary provider of PD care in mainland China (*P*=.03).

### Clinical Conditions Impacting PD Decision

Data from the 788 submitted surveys showed that besides chronic and acute renal failure, secondary hospitals tended to apply less PD compared to tertiary hospitals for acute congestive heart failure (*P*=.05), electrolyte (*P*=.03), acid-base disorders (*P*=.02), and drug intoxications (*P*=.10; Figure 1C). Between the secondary and tertiary hospitals, there was no significant difference in factors hindering PD decision when coming across clinical situations, such as extensive adhesion of the peritoneum (776/788, 98%), unrepaired hernia (721/788, 91%), and severe skin disease (671/788, 85%).

### Treatment Module of PD

The most common PD type was continuous ambulatory peritoneal dialysis, which was reported by 98% (772/788) of the respondents. It was followed by intermittent peritoneal dialysis 69% (543/788). The overall reported nocturnal intermittent peritoneal dialysis, continuous cycling peritoneal dialysis, and tidal peritoneal dialysis were 31% (244/788), 28% (220/788), and 15% (118/788), respectively. Automated PD was available in 32% (37/115) of the secondary facilities and 55% (347/631) of the tertiary facilities by the end of October 2020 (Figure 1C). A combination of hemodialysis and PD was reported by 11 centers; daytime ambulatory PD was reported by 23 centers, and a combination of manual continuous ambulatory peritoneal dialysis and automated PD was reported by 1 center.

### Management of PD Centers

Among the secondary centers, 17% (20/115) reported that there were no established management rules for the PD center, while this number was 7% (44/631) in tertiary hospitals (*P*=.002).

About 19% (22/115) of the secondary hospitals did not have specialized PD medical records for each patient, and this was 11% (69/631) in the tertiary hospitals (*P*=.01).

**Figure 2:**
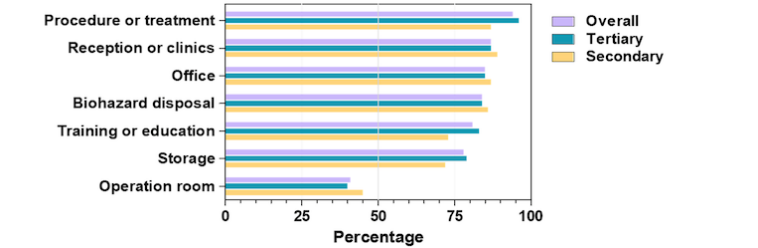
Proportion of hospitals having functional areas designated for peritoneal dialysis use.

More than 80% of the centers reported separate functional areas designated for PD care, including areas for reception, procedure or treatment, biohazard disposal, and staff office (Figure 2), with no significant statistical difference between the secondary and tertiary hospitals. Compared to the secondary hospitals, tertiary centers were more likely to have designated areas for training or patient education (*P*=.01), storage (*P*=.09), and procedures (*P*<.001). Only 40% (289/722) of the PD centers had operation rooms exclusive for dialysis access, and there was no discernible difference between the secondary and tertiary centers.

### PD Catheter Implantation and Care

About 96% (608/631) of the participating tertiary hospitals performed implantation surgery for PD catheters, which involved 78% (515/662) of the survey participants; this was performed in 86% (99/115) of the secondary centers (vs tertiary centers; *P*<.001) and by 67% (85/126) of physicians in the secondary hospitals (vs tertiary hospitals; *P*=.01). Assessment and patient education were conducted before the implantation surgery in 100% of the 631 tertiary hospitals and 94% (108/115) in the secondary centers. Among the hospitals performing PD catheter implantation, 100% (746/746) provided preoperative assessment and patient education. Cephalosporin of the first or second generation was commonly used right after the surgery, accounting for 48.7% (384/788) of the antibiotics used. Other antibiotics included third or fourth-generation cephalosporin (72/788, 9.1%), quinolone (10/788, 1.3%), and aminoglycosides (4/788, 0.5%). About 40.4% (318/788) of the participants reported using antibiotics other than the ones mentioned above or not using them. There was no significant difference in postoperative antibiotic use between the tertiary and secondary hospitals.

There was a slight but significant difference in the frequency of replacing the external tube that connects to the PD catheter (*P*=.04). Among the secondary hospitals, 82% (94/115) changed the connecting tube every 6 months, and 15% (17/115) did so every 3 months; this was 90% (568/631) for every 6 months and 7% (44/631) for every 3 months in the tertiary hospitals. A few centers reported replacement frequency at every 9 months (about 2%) or every 12 months (<1%) from both secondary and tertiary hospitals.

### Management of PD Patient Follow-Up

**Figure 3:**
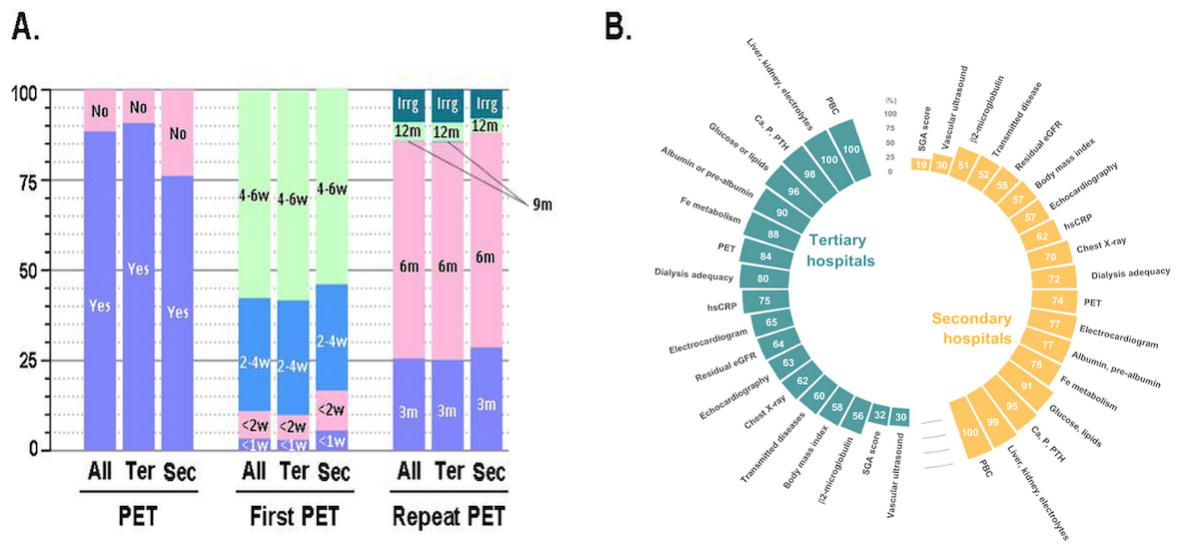
Follow-up Items for peritoneal dialysis. (A) Availability of peritoneal equilibrium test (PET) and the time it was performed (ter: tertiary hospital; sec: secondary hospital; w: week). (B) The follow-up items regularly applied in secondary and tertiary hospitals for patients on peritoneal dialysis. Ca: Calcium; eGFR: estimated glomerular filtration rate; Fe: Ferrum; hsCRP: high-sensitive C-reactive protein; P: Phosphorus; PBC: peripheral blood cell; PTH: parathyroid hormone; SGA: subjective global assessment.

The use of PET to assess the function of the peritoneum was reported in 91% (574/631) of the tertiary hospitals and 76% (87/115) of the secondary hospitals (*P*<.001; Figure 3A). Around 58% (454/788)of the first PETs were performed within 4-6 weeks after initiation of PD, and 31% (247/788) conducted the first PET between 2-4 weeks overall in tertiary and secondary hospitals. PET was repeated every 6 months in 60% (475/788) of the PD centers, and another 25%-29% (166/662 for tertiary hospitals and 36/126 for secondary hospitals) of the centers had repeat PET every 3 months (Figure 3A).

For PD long-term care, the most common follow-up interval was every month (385/788, 49%), followed by every 3 months (226/788, 29%), irregular (85/788, 11%), every 2 months (74/788, 9%), and every 6 months (18/788, 2%).

For the laboratory tests and imaging examinations, peripheral blood count was considered for each patient (788/788, 100%); blood biochemistry panel (ie, parameters for liver enzymes, kidney function, and electrolytes; 788/788, 100%); serum calcium, phosphate, intact parathyroid hormone (772/788, 98%); and glucose and lipid profiles (748/788, 95%) were routinely followed. Parameters for iron metabolism (*P*=.002), nutrition status (ie, albumin and pre-albumin; *P*<.001), and inflammation biomarker high-sensitive C-reactive protein (*P*=.003) were more likely to be followed up in the tertiary hospitals compared to the secondary hospitals (Figure 3B). Transmitted disease panel, BMI, serum β_2_-macroglobulin, chest x-ray, electrocardiogram, and cardiovascular ultrasound were also checked in both tertiary and secondary centers, with checking rates ranging from 30% (236/788) to 67% (527/788). PET (*P*=.01), PD adequacy test (*P*=.06), and assessment for residual kidney function (*P*=.04) were administered more in tertiary hospitals than in secondary hospitals.

### PD Complication Management

**Figure 4:**
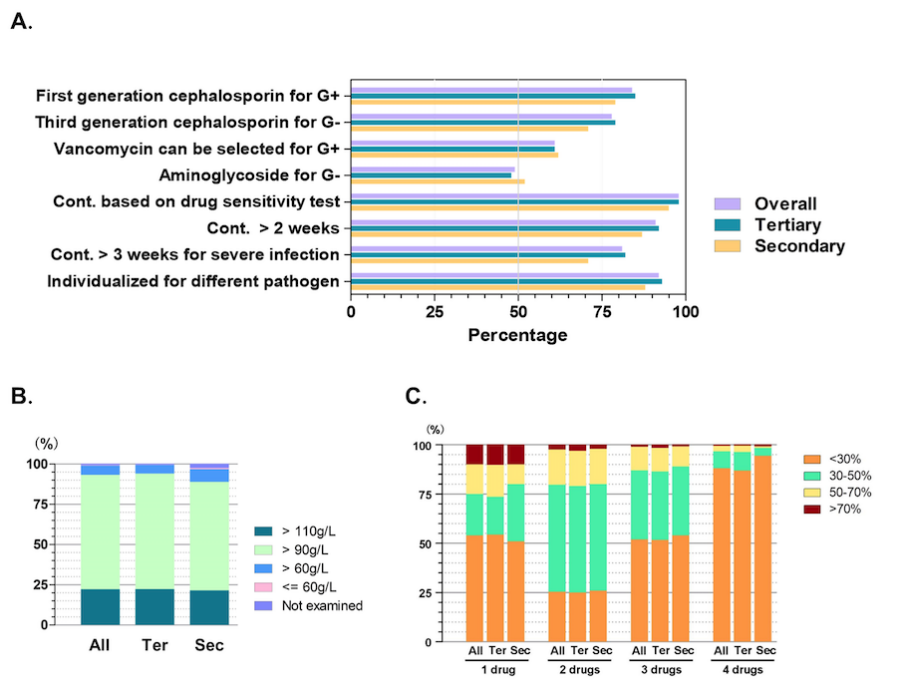
Complication management. (A) Antibiotic choice for peritonitis (G+: gram-positive; G–: gram-negative; Cont: continued). (B) Hemoglobin level in patients on peritoneal dialysis. (C) Number of antihypertensive drug used (ter: tertiary hospital; sec: secondary hospital).

For infectious peritonitis, about 84% (659/788) of the participants chose first-generation cephalosporin for gram-positive bacteria, and 78% (612/788) chose third-generation cephalosporin for gram-negative pathogens (Figure 4A). Vancomycin was also commonly used for the treatment of gram-positive peritonitis, and aminoglycoside was used for gram-negative peritonitis. For severe infection, 29% (33/115) of the secondary hospitals did not continue antibiotic therapy longer than 3 weeks, compared to 18% (114/631) of the tertiary centers (*P*=.005). Awareness of applying drug sensitivity tests and individualized use of antibiotics was more than 92% (725/788) among the participants.

In the secondary hospitals, 89% (112/126) of the patients on PD had hemoglobin levels greater than 90 g/L, compared to 94% (623/662) of them in the tertiary centers (Figure 4B).

The majority of patients on PD took 1-3 antihypertensive drugs, with a total of 97% (764/788; 95% CI 95%-98%) of the respondents prescribing 1-3 drugs for more than 30% of the patients (Figure 4C). A combination of 2-3 antihypertensive medicines was more common than using a single drug (677/788, 86%, 95% CI 83%-88% vs 362/788, 46%, 95%CI 43%-50%; *P*<.001) or ≥4 drugs (vs 95/788, 12%, 95%CI 10%-14%; *P*<.001).

## Discussion

To our knowledge, this survey was the first nationwide study focusing on the current situation of PD management and PD practitioners’ concepts and behavior in mainland China [1,12,14]. It covered about 80% of PD centers and nearly 90% of the estimated number of current patients on PD from all provincial-level areas, according to previously published registry data [9,13], as well as about 70% of PD centers and 80% of patients on PD, according to unpublished CNRDS 2021 data. The respondents who filled the survey were mostly physicians in charge of the PD centers or some chief nurses who were well knowledgeable about the PD center operations. The big number of the returned surveys and the personnel responding to the questionnaires suggested a faithful reflection of the real situations that may not be accurately captured or reported by the registries. The participating numbers of tertiary and secondary hospitals were about 20% and 1% of the total number of tertiary and secondary hospitals in mainland China, respectively, according to the 2020 China Health Care Development Statistics [4]. In this study, we reported that PD care was provided mostly by tertiary hospitals in mainland China, and about 5% of patients were managed by secondary hospitals. We found that tertiary hospitals had a more complete infrastructure of PD centers, performed more operations for PD catheter placement, treated more PD indication–related acute diseases, and conducted more assessments for peritoneal membrane characteristics and dialysis adequacy than the secondary hospitals. This study also showed that current concepts of the goals for long-term PD complication management were adequately adopted by health care providers (HCPs) in both tertiary and secondary hospitals, with a relatively similar compliance rate of the follow-up items.

Our study showed a couple of differences in PD care management between the secondary and tertiary hospitals. In the secondary hospitals, a lack of designated infrastructure for PD patient education was common, and the PD catheter placement procedure was significantly less performed. This discrepancy could be explained by the lack of clinical experience and human power as well as low financial incentive for PD catheter implantation. To overcome these barriers, it is necessary to implement a well-designed and feasible PD program for catheter implantation. Sufficient surgical training, a dedicated team and time, and protected reimbursement from payers would help to achieve this goal. Complex and more advanced PD treatment modules, such as cycler-facilitated nocturnal intermittent peritoneal dialysis or tidal peritoneal dialysis as well as a combination of hemodialysis and PD, were significantly less reported by secondary hospitals. We speculate that, along with the development of digital tools for PD patient management, more available web-based patient education resources, more use of PD cycler, and the shifting of reimbursement policy toward lower tiers of cities and health care facilities, more patients with incident dialysis will choose PD and more patients on PD will migrate to secondary and even primary health care centers for chronic PD management. In that regard, the proportion of patients on PD in rural and lower-tier facilities is expected to increase rapidly in the near future, which advocates strengthened implementation of standardized PD quality management and control at high priority.

Adequate patient follow-up scheme and good execution are necessities for quality PD care [16]. In China, a usual PD outpatient follow-up routine includes HCPs’ tracking of the daily records of PD exchanges, measurement of blood pressure, prescriptions for examinations, and medication adjustment [17]. Our survey results showed that more than 85% of patients on PD were regularly followed every 1 to 3 months, and almost every patient was checked for anemia (low hemoglobin) and mineral bone markers (ie, calcium, phosphorus, and parathyroid hormone), which was similarly complied according to multiple international guidelines and recommendations [18-20]. These proportions were higher than that reported in China Kidney Disease Network 2016 Annual Data Report, which was around 90% [8]. The difference may be explained as follows: our survey covered wider regions and more patients on PD, whereas the China Kidney Disease Network Report used data sources from claim databases and included primary hospitals. Nonetheless, thanks to the continuous effort of improving health care accessibility and affordability from the government at all administrative levels, results from these 2 studies indicate that patients with ESKD are getting an adequate standard of care in mainland China.

In our survey, there were about 10% (10,150/101,537) of patients on PD whose hemoglobin level was below 90 g/L, the level at which rescue therapy for anemia should be considered [21]. These data were also similar to the data recently reported by the US Renal Data System [16]. Multiple guidelines recommend treating chronic kidney disease anemia to the hemoglobin level of 110-120 g/L [21,22] or around 110 g/L [18,19]. In our study, about 22% (175/788) of the respondents reported hemoglobin levels of their patients on PD as above 110 g/L, with no difference between the secondary and tertiary hospitals, indicating that further clinical effort may be needed to reduce the anemia burden in patients on PD. Multidrug therapy is usually required and recommended for the control of blood pressure in patients with chronic kidney disease worldwide [23-25]. In PD, antihypertensive treatment is dependent on not only the drug administration but also fluid volume control. Our study showed that 86% (87,321/101,537) of patients on PD took 2-3 antihypertensive medicines. Future studies on the relationships among blood pressure level; types of antihypertensive drugs; volume status; and other factors, such as retaining of residual kidney function, longevity of peritoneal membrane function, anemia treatment regimen, cardiovascular outcome, patient compliance, and health economics would provide guidance to improve PD outcomes as well as goal-directed value-based PD care [17,26].

Assessment for peritoneal membrane characteristics is helpful for appropriate PD prescription. International PD guidelines consistently recommended performing the first PET at 4-6 weeks after PD initiation and having it reevaluated every 6-12 months [27-30]. In our study, PET was not performed in 9% (57/631) of the tertiary hospitals and 24% (28/115) of the secondary hospitals. About 42% (334/788) of the first PETs was performed within 4 weeks after initiation of PD. Moreover, about 26% (202/788) of the respondents repeated PET every 3 months. All these indicate that Chinese HCPs use PET more frequently than those tests commonly recommended by different guidelines, suggesting that in-depth education about PET should be enhanced.

Infectious peritonitis or PD catheter–related infection is the major PD complication that affect long-term PD survival and require immediate empirical treatment with antibiotics and systemic long-term management [31,32]. In China, most participating hospitals implemented the same recommendations of the guideline of International Society for Peritoneal Dialysis [33] for the management of peritonitis in PD. Our survey revealed that 92% (726/788)of PD HCPs acknowledged the values of pathogen detections and drug sensitivity tests, continued antibiotic treatment for 2-3 weeks, and individualized antibiotic regimen for PD-related peritonitis [30,33]. For initial empirical therapy, 84% (659/788) of respondents chose first-generation cephalosporins for gram-negative pathogens, and 78% (612/788) chose third-generation cephalosporins for gram-negative pathogens. Besides the cephalosporins, vancomycin was also chosen for gram-positive pathogens by 61% (481/788) of the respondents and aminoglycoside for the gram-negative pathogens by 49% (386/788). These patterns of choice were in line with multiple guidelines or recommendations regarding PD-related peritonitis [29,30,33].

In our study, 95% (96,460/101,537) of the patients on PD were managed by tertiary hospitals, despite the fact that the number of secondary hospitals in China is about 5 times that of the tertiary hospitals. Although our survey covered a much larger PD patient population, it was likely that our data were skewed toward tertiary facilities. It is also possible that some transferring of patients on PD overlapped, and data were not completely aggregated for analysis in our study.

It is known that the quality of ESKD care is influenced by socioeconomic status and geographic regions [34]. Except for the number of responded hospitals and patients on PD, this study did not find significant regional differences in the answered items listed in the questionnaire. The dominance of patients in tertiary hospitals in this study might contribute to the diminished possible regional discrepancy because most of the tertiary hospitals similarly follow the SOP for PD, despite being from different regions. Due to the infrastructure inadequacy, there were still 13% (94/722) of the PD centers that did not have a dedicated PD reception area; 19% (137/722) had no PD training room, 22% (159/722) had no separated PD storage room, and only 41% (296/722) had their unique PD operation room. Furthermore, 9% (65/722) of PD centers had not established local SOP, and 12% (87/722) had no specific PD-related health records. These need to be improved in the future for sustainable growth of PD along with high-quality standards of care.

This study did not limit the qualification of participants from each hospital and used voluntary responses, and this could cause selection bias. In addition, disposing bias might be introduced during the process of aggregating data from the same facility. More studies on the relationship between PD clinical outcomes and hospital quality management can be more instructive for health care policy makers. Future studies addressing improved prognosis of patients on PD through strengthened measures will require longitudinal analyses using both registry and survey data.

In summary, this first national survey provided data reflecting the current status of PD center management in mainland China. It also collected HCPs’ concepts for the routine practice of PD patient management. The discrepancy of PD care between the secondary and tertiary hospitals may guide future iteration of the SOP for PD and quality management, targeted HCP training for PD, as well as value-oriented patient education. To a broader extent, providing a better goal-oriented PD care would help to reduce the substantial health care costs and improve the quality of life of patients with ESKD.

## Abbreviations

CNRDS: Chinese National Renal Data System
ESKD: end-stage kidney disease
HCP: health care provider
PD: peritoneal dialysis
PET: peritoneal equilibrium test
SOP: standard operating procedure
